# Analysis of the Variability of Therapeutic Indications of Medicinal Species in the Northeast of Brazil: Comparative Study

**DOI:** 10.1155/2018/6769193

**Published:** 2018-04-23

**Authors:** Julimery Gonçalves Ferreira Macedo, Irwin Rose Alencar de Menezes, Daiany Alves Ribeiro, Maria de Oliveira Santos, Delmacia Gonçalves de Mâcedo, Márcia Jordana Ferreira Macêdo, Bianca Vilar de Almeida, Liana Geraldo Souza de Oliveira, Catarina Pereira Leite, Marta Maria de Almeida Souza

**Affiliations:** ^1^Department of Biology, Vegetal Ecology Laboratory, Regional University of Cariri, 63105-000 Crato, CE, Brazil; ^2^Department of Biological Chemistry, Laboratory of Pharmacology and Molecular Chemistry Regional University of Cariri, 63105-000 Crato, CE, Brazil

## Abstract

**Ethnopharmacological Relevance:**

This study aims to evaluate the versatility of these species and their agreement of use and/or the informants' knowledge and verify the variability of the information on the indicated medicinal species in comparison to other species from northeastern Brazilian areas.

**Materials and Methods:**

Ethnobotanical information was acquired through interviews with 23 residents of the Quincuncá community, northeastern Brazil. From the obtained data, a comparative analysis of the therapeutic indications with other 40 areas in different biomes was conducted. For that, the relative importance index and informant consensus factor were calculated and compared to other indices evaluated in the literature.

**Results:**

A total of 39 medicinal species were cited and twenty-six species showed similarities among their therapeutic indications; however, species as* Geoffroea spinosa*,* Lantana camara*, and others can be highlighted, present in community disease indications that were not verified for other areas.* Myracrodruon urundeuva*,* Mimosa tenuiflora*,* Stryphnodendron rotundifolium*, and* Amburana cearensis* had the greatest versatility. In the Quincuncá community, medicinal species were indicated for 49 diseases, which were grouped into 15 categories of body systems.

**Conclusion:**

This study shows the presented divergence in relation to their therapeutic use; in this point, these divergences reinforce the importance of pharmacological research.

## 1. Introduction 

The use of medicinal plants for therapeutic purposes is one of the oldest health treatment practices [1, 2]. In Brazil, even with the advancement of the pharmaceutical industry, the use of medicinal plants is very recurrent and can be influenced by the high cost of medicines, economic issues, the difficult access to consultations by the Unified Health System (SUS), locomotion difficulties for those living in rural areas, the easy access to plants, or the current trend of using natural resources as an alternative to synthetic medicines [3, 4].

Many medicinal species stand out for their curative potential [5] as they are widely known and used in popular medicine; these species contemplate the treatment and/or cure of various bodily systems mainly related to the respiratory, digestive, genitourinary disorders, and undefined conditions [6–8].

Many medicinal plants that show an indication for medicinal use based on knowledge, and which are present in ethnobotanical surveys, have already had their therapeutic indications proven through pharmacological bioprospecting studies. Among the main confirmed activities are: antibacterial [9–11] antinociceptive, anti-inflammatory, anti-oxidant [12–14] and cicatrizant activity [15].

In recent years there have been an increasing number of ethnobotanical studies in various parts of the world. Due to the biome diversity present in Brazil, in addition to the difficulty in accessing the primary healthcare system, ethnobotanical and ethnopharmacological studies represent an important mechanism for therapeutic proposal in various areas of the country. These studies however present a wide variety in the use and therapeutic indications of medicinal species. According to Ribeiro et al. (2014), the variation in therapeutic indications legitimized by the relative importance of the species may be related to the different types of diseases and bodily systems that a given community needs to treat [16]. Often in a community, the same plant is used for various types of diseases and/or symptoms, yet in another community with geographical proximity, it is almost unknown for its therapeutic properties. In this sense, this study aimed to evaluate the versatility of species and the agreement of use and/or knowledge of the informants of the Quincuncá community and to verify the variability of the information of the indicated medicinal species against other areas of northeastern Brazil.

## 2. Material and Methods

### 2.1. Selection of Medicinal Species

Selection of the species occurred in the Quincuncá community, located approximately 11 km from the city Farias Brito (6°55′50′′S and 39°33′56′′W). The predominant climates are tropical hot semiarid and tropical cold semiarid, with average temperatures ranging from 26°C to 28°C. The annual precipitation is around 967.8 mm, with rainfall concentrated in the months of January to April [17, 18]. It is also characterized by vegetation from the Caatinga, Cerrado, and Subdeciduous forests, reliefs from Sertaneja depressions, and residual massifs, with an altitude of 320 m [18].

For the selection of the species, 23 key informants were interviewed, of which 13 were women and 10 were men, between the ages of 27 and 89 years. The study was conducted with key informants that were selected using the “snowball” technique adapted to Albuquerque et al. (2010) [19]. The information about the medicinal plants used and their different therapeutic uses was obtained from semistructured interviews based on a standardized form [20]. The interviews were only possible with the informants reading, permission, and signing of the Term of Free and Informed Consent [21]. This research was approved by the Ethics Committee of the Regional University of Cariri, under legal number 1.654.716.

The species cited in the interviews had their plant material collected, stored, and handled according to conventional herbalism techniques [22]. Subsequently, all the collected material was taken to the Plant Ecology Laboratory of the Regional University of Cariri for exsiccating preparation. The identification of the medicinal species took place at the Caririense Dárdano de Andrade-Lima Herbarium of the Regional University of Cariri, by means of specialized literature, comparison of the deposited exsiccates, and analyses by taxonomists with the testimonial material being incorporated into the collection of the above herbarium. The authorization to collect the botanical material was provided by the Authorization and Biodiversity Information System (SISBIO) of the Brazilian Institute of Environment and Renewable Energies Natural Resources (IBAMA), registered under number 55704-1.

### 2.2. Survey of Ethnobotanical Studies for Comparative Analysis

For comparative analysis, we conducted an active search in the Web of Science, PubMed, Science Direct, Google Academic, and Medline databases in the last 10 years using terms such as ethnobotany, ethnopharmacology, popular knowledge, medicinal plants, northeastern Brazil. Articles that met the inclusion criteria, scientific articles published in specialized indexed journals, articles published in the time window between the years 2006 and 2016, articles within the area of the Brazilian northeast, and articles that had at least one of the species indicated by the Quincuncá community, were selected.

### 2.3. Data Analysis

From the information collected in the community, the relative importance (RI) was calculated, based on Bennett and Prance (2000), with “2” being the maximum value that a species can acquire [23]. The calculation is done according to the formula: RI = NBS + NP, where NBS = NBSS/NBSVS and NP = NPS/NPVS. RI is the relative importance, NBS is the number of bodily systems, NBSS is the number of bodily systems treated by a particular species, and NBSVS is the total number of bodily systems treated by the most versatile species. The NP is the number of properties, NPS is the number of properties attributed to a particular species, and NPVS is the total number of properties attributed to the most versatile species [24, 25]. The RI^1^ was determinate for information collected in the community and RI^2^ was used by arithmetical media from relative importance collected in literature. However, for the articles selected in the survey, the relative importance index was calculated considering only the native species and using the medical indications reported in each study.

The Informants Consensus Factor (ICF) was calculated according to Troter and Logan (1986), using the formula: ICF = *nur* − *na*/*nur* − 1, where ICF is the Informants Consensus Factor, (*nur*) is the number of usage citations in each category, and (*na*) is the number of species used in each category [26]. The maximum value obtained by the ICF is 1. For the work obtained from the literature review, this index was calculated in an adapted manner taking into account the fact that each indication within the studied community was equivalent to a citation, with all the communities being grouped to reach the ICF value.

Therapeutic indications were assigned by establishing therapeutic indications based on body system categories as described in the International Statistical Classification of Diseases and Related Health Problems (ICD-10) proposed by the World Health Organization (WHO) [27]: Undefined Affections and Aches (NDDP), Mental and Behavioral Disorders (MBD), Disease of the Endocrine Glands, Nutrition and Metabolism (DEGNM), Infectious and Parasitic Diseases (IPD), Diseases of the Skin and Subcutaneous Tissue (DSSCT), Diseases of Blood and Hematopoietic Organs (DBHO), Diseases of the Musculoskeletal System and Connective Tissue (DMSCT), Injuries, Poisonings and other Consequences of External Causes (IPOCEC), Neoplasms (N), Diseases of the Circulatory System (DCS), Disorder of the Digestive System (DDS), Disorder of the Genitourinary System (DGS), Diseases of the Nervous System (DNS), Respiratory System Disorder (RSD), Disorders of the Visual Sensory System-Eyes (DVSS-E), and Disorders of the Auditory Sensory System-Ears (DASS-E).

## 3. Results and Discussion

### 3.1. Survey of Medicinal Species

In the bibliographical survey, 350 articles were identified. After applying the process of inclusion and exclusion of duplicate articles, 40 articles that presented therapeutic indications of the species common to the Quincuncá community and the different areas in the Northeast of Brazil, Paraíba, Pernambuco, Ceará, Piauí, Rio Grande do Norte, Bahia, and Maranhão, with Caatinga, Cerrado, Carrasco, Atlantic Rainforest, and Amazonian vegetation, were selected [6, 7, 16, 28–64].

In the community study, 39 native species belonging to 17 families and 36 genera (Table 1) were raised, from these, six (*Myracrodruon urundeuva*,* Amburana cearensis*,* Anadenanthera colubrina*,* Libidibia ferrea*,* Ximenia americana*, and* Ziziphus joazeiro*) appeared in more than 50% of the communities, indicating broad knowledge and/or use of these species in the Brazilian northeast. It is worth mentioning that* Geoffroea spinosa* was present in only one community. The results show an expressive number of native medicinal species when compared to other ethnobotanical studies in the northeast, with values varying from 3 to 84 species [31, 54]. These numbers are considered quite superior when they include exotic species, presenting a wealth of up to 187 medicinal species [65].

The families with the highest number of species were Fabaceae (15 spp.), Rubiaceae (4 spp.), and Euphorbiaceae (3 spp.). The Fabaceae family is considered to be the largest holder of the number of species in the state of Ceará, represented by 521 species [66], and also presents the highest species representativeness in ethnobotanical studies in the Brazilian Northeast [38, 44, 67]. For the genera, we have* Croton* with three species and* Mimosa* with two and the others presented only one species. Castro et al. (2005) state that plants belonging to the* Croton* genus are rich in essential oils with wide biological activity [68].

Regarding the used plant parts for the preparation of medicines, the Quincuncá community inhabitants indicated 10 different types, while in other areas the inhabitants indicated 12 (Table 1). Stem bark, leaf, stem inner bark, and root stood out for all areas studied. Stalk, fruit, flower, root-tuber, latex, resin, seed, and the whole plant were indicated in less than 30% of the areas. Most of the communities analyzed are located in the Caatinga, which may have increased the amount of stem bark indication as these are available throughout the year [47, 69]. Regarding the preparation method, decoction stood out, with this result being similar to the majority of other studies carried out in the Brazilian Northeast, where according to Ribeiro et al. (2014) [16] and De Moraes Rego et al. (2016) [70] there is a predominance in the use of teas.

### 3.2. Therapeutic Indications of Medicinal Plants

For the studied community, among the 39 cited species, 28 (71.79%) are employed for more than one health problem, while 11 (28.20%) have only one therapeutic utility. Together, these species were indicated for 52 medicinal purposes.* Myracrodruon urundeuva* and* Mimosa tenuiflora* presented the highest number for variation use, ranging from 1 to 8 indications. In the survey of the communities in the Northeast of Brazil, a variation from 1 to 72 medical purposes and a total of 210 health problems were reported. For example,* Myracrodruon urundeuva* obtained between 1 and 20 indications in 31 communities [59, 63], with an average of 5.74 disease per community;* Amburana cearensis*, in 26 communities, obtained from 1 to 17 indications [36, 44], with an average of 5.5;* Stryphnodendron rotundifolium *in 6 communities, obtained from 3 to 10 indications [32, 35], with an average of 6. These data show the great variability in use and/or knowledge of the species by the communities.

It was observed that, within 26 species, at least one therapeutic indication equal to the medical uses in other areas was reported showing a significant agreement in the indications of use; however, within 13 species, divergence occurred in relation to their therapeutic uses. Among the species that presented the same therapeutic indications,* Myracrodruon urundeuva* and* Amburana cearensis* can be highlighted, having been cited in more than 60% of the communities due to their uses directed towards general inflammation and respiratory diseases, respectively. These results may be justified by the availability of their resources (stem, bark), since the species mentioned above are widely distributed and very characteristic of Caatinga areas. By analyzing the availability of resources in a given community, de Albuquerque and Andrade (2002) realized that the most important species are usually those that offer their products continuously [69]. Plants that offer their resources for a few months, especially in rainfall, are rarely mentioned in the community and rarely used, and this is confirmed by their relative importance [71]. Another assumption is that communities have a high degree of confidence in the healing power of these species, so their therapeutic indications are widespread.

Among the species that diverged regarding therapeutic uses,* Geoffroea spinosa*,* Lantana camara*,* Senegalia tenuifolia*, and* Licania rigida* can be highlighted. The species* Geoffroea spinosa* was cited for fever, dysentery, and snakebite in the studied community; however, it was cited only for the treatment of anemia in other studies. Meanwhile,* Lantana camara* was indicated for the treatment of hypertension by the studied community, yet in other areas, it is indicated mainly in the treatment of respiratory diseases such as cough and flu and also for rheumatism.* Senegalia tenuifolia* was recommended for diabetes, cancer, and hypertension in the studied community, with these indications not being found in other areas, whereas it was indicated for spine, influenza, cicatrizing, and rheumatism in other areas.

The species* Licania rigida* was indicated for uterine and ovarian inflammation in the studied community; however, it stands out in other ethnobotanical surveys for the treatment of diseases related to Diseases of the Endocrine Glands, Nutrition and Metabolism such as diabetes, cholesterol, weight loss, and hypoglycemia, also being reported for diseases such as stomach ache, general inflammation, diarrhea, kidneys, dandruff, ringworm, and itching. In this case, there may be a common use, since both in the survey and in the indication by the studied community there is an agreement for inflammatory processes. The divergence regarding therapeutic use observed in this work may be explained, in part, due to the nonavailability of natural resources, the lack of knowledge of these species for therapeutic indication, or the existence of other species that are used to treat diseases present in the body system.

Most species were mentioned by both women (36) and men (31); however,* Syagrus oleracea, Jacaranda jasminoides, Erythrina velutina, Senegalia tenuifolia, Bredemeyera brevifolia, Licania rigida*, and* Lippia microphylla* were reported only by females and* Bromelia laciniosa* and* Guettarda angelica* were only reported by the male sex. This exclusivity may be related to species indicated for sex-specific diseases such as ovarian inflammation for women and prostate problems for men.

### 3.3. Versatility of Medicinal Species

Of the medicinal species in the community, seven have great relative importance (RI > 1) (Table 2):* Myracrodruon urundeuva* (RI^1^ = 1.87)*, Mimosa tenuiflora* (RI^1^ = 1.87),* Myroxylon peruiferum *(RI^1^ = 1.80),* Commiphora leptophloeos* (RI^1^ = 1.45),* Coutarea hexandra* (RI^1^ = 1.30),* Poincianella pyramidalis* (RI^1^ = 1.10), and* Ximenia americana* (RI^1^ = 1.02). The other species obtained lower values, ranging from 0.32 to 1.0. Of these species,* Myracrodruon urundeuva *(RI^2^ = 1.35) and* Ximenia americana *(RI^2^ = 1.29) also presented the greatest versatility in other areas, together with* Stryphnodendron rotundifolium *(RI^2^ = 1.47),* Amburana cearensis* (RI^2^ = 1.33)*, Bauhinia cheilantha* (RI^2^ = 1.17)*, Hymenaea courbaril* (RI^2^ = 1.15),* Croton heliotropiifolius *(RI^2^ = 1.13)*, Ziziphus joazeiro* (RI^2^ = 1.05), and* Operculina macrocarpa* (RI^2^ = 1.04), totaling nine species, with the others ranging from 0.30 to 1.0 (Table 2).

The species* Commiphora leptophloeos* (RI^1^ = 1.45/RI^2^ = 0.51),* Croton heliotropiifolius* (RI^1^ = 0.45/RI^2^ = 1.07),* Bauhinia cheilantha* (RI^1^ = 0.32/RI^2^ = 1.17),* Erythrina velutina* (RI^1^ = 0.32/RI^2^ = 0.99),* Geoffroea spinosa* (RI^1^ = 0.97/RI^2^ = 0.30),* Mimosa tenuiflora* (RI^1^ = 1.87/RI^2^ = 0.68),* Myroxylon peruiferum* (RI^1^ = 1.80/RI^2^ = 0.60),* Stryphnodendron rotundifolium *(RI^1^ = 0.32/RI^2^ = 1.47),* Bredemeyera brevifolia* (RI^1^ = 0.77/RI^2^ = 0.29),* Myracrodruon urundeuva* (RI^1^ = 1.87/RI^2^ = 1.35), and* Coutarea hexandra* (RI^1^ = 1.30/RI^2^ = 0.57) presented significant disagreement in the number of citations indicated by the Quincuncá community when compared to the number of citations indicated in the survey for other regions of the northeast. In this sense, this divergence can be considered important, since it indicates the need for biological and pharmacological studies that validate its activities. However, from the ecological point of view, this divergence also deserves special attention, since they demonstrate areas where greater extractivism occurs due to medicinal use.

Most of the cited species, circa 59%, presented a RI^2^ value higher than RI^1^ calculated for the study area. Of the species,* Stryphnodendron rotundifolium* presented the highest mean RI, with an interval range of 0.97 to 2.00 and* Bredemeyera brevifolia* the lowest mean, with RI^2^ = 0.29 and an interval from 0.17 to 0.44. Species such as* Genipa americana* (RI^2^ = 0.66) and* Lantana camara* (RI^2^ = 0.58) that presented low RI averages can be highlighted as having the maximum RI value in ethnobotanical surveys in the northeast, as demonstrated in the works of Cordeiro and Félix (2014) [57] and Guerra et al. (2016) [64], respectively.

Around 80% of the species obtained relative importance values <1. Although some of these species present values that are considered low, with few therapeutic indications and bodily systems, they should not be considered of less therapeutic potential.* Bauhinia cheilantha*,* Genipa americana*,* Erythrina velutina*, and* Solanum paniculatum* showed in the studied area RI^1^ = 0.32, while for other areas they presented relative importance values of 2.0, 2.0, 1.85, and 1.75 [16, 33, 52, 57], respectively, demonstrating greater indications of use covering a greater number of body systems.

*Stryphnodendron rotundifolium* proved to be the most important species for the analyzed communities; it appears in only six communities, yet it presented the highest relative importance RI^2^ = 1.47. It is traditionally used by northeastern Brazilian populations for various types of inflammation, such as uterine, general, skin, and throat inflammation, as well as being potentially indicated as a cicatrizant and for the treatment of cancer [72]. It is considered a promising species for bioprospecting, since it has already demonstrated activity as an antioxidant [73], leishmanicidal and trypanocidal activity [74], and antimicrobial activity [75, 76].

*M. urundeuva* appears in this study as being reported for a greater number of diseases and bodily systems, giving it a greater versatility of use (RI^1^ = 1.87). This was indicated for the treatment of wounds, inflammation, influenza, menstrual bleeding, anemia, healing, and uterine inflammation, covering five bodily systems where the system related to injuries, poisonings, and other consequences of external causes stands out (5 citations) (Table 2). This species seems to have a good distribution and reports of medicinal uses, since it appears in almost all the areas analyzed in the survey being indicated for the cure and/or treatment of 72 diseases, where the most outstanding are inflammation (25), healing (15), gastritis (8), uterine inflammation, wounds, and cough (7 each), related to 13 body systems, being the most comprehensive NDDP (37), DGS (34), DDS (30), IPOCEC (26), and RSD (20).* M. urundeuva* showed the second highest mean relative importance (RI^2^ = 1.35) for the communities, with values varying from 0.40 to 2.0, and in more than 75% of these communities the RI was >1. Pharmacologically, this species already has scientific confirmation for a variety of activities, among which is its antileishmaniasis [77], antibacterial [78], antiviral [79], antiulcer [80], and anti-inflammatory activity [81], which in part is attributed to the presence of tannins, flavonoids, and dimeric chalcones isolated from the bark, proving to be an effective analgesic in wound healing, when used in animal models [82].

*Mimosa tenuiflora* also presented the greatest versatility of use (RI^1^ = 1.87) for the studied community. In some communities this species is also of relative importance >1, with values of 1.16, 1.23, and 1.32 in studies by Leite et al. (2015) [30], Roque et al. (2010) [53], and Oliveira (2015) [29], respectively. This is a species widely distributed in the Caatinga, typical of the Brazilian semiarid, and appreciated for its foraging potential, energy, and medicinal properties [83]. Some research also reports it as being used for religious purposes and mystical cults, for presenting psychotropic properties due to the presence of alkaloids [71, 84]. Regarding its therapeutic indications,* M. tenuiflora* was cited mainly for inflammation, wounds, and tooth ache; results are consistent with the indications in other northeast areas with indications for wound (9), inflammation (5), toothache, and healing (4 each), with the IPOCEC, NDDP, and DDS being the most recurrent systems. Among the activities already proven for this species, the cicatrizant activity [85] and anti-inflammatory and antinociceptive activity [86] can validate the popular use of its mentioned indication. As for its chemical components, it possesses tannins, flavones, catechins, leucoanthocyanins, and saponins [87]. In pharmacological studies carried out in Mexico, the authors point out to tannins as one of the compounds responsible for the biological activities of the plant stem [85].

*Myroxylon peruiferum* obtained the second highest relative importance value (RI^1^ = 1.80) for the studied community, with the same not occurring in the other regions, where it was reported in four articles [7, 53, 56, 62] and obtained a RI^2^ = 0.60. The most cited therapeutic indications for* M. peruiferum* were the liver and kidneys (2 each), in addition to influenza, intestine, prostate, hypertension, indigestion, and stomach with a citation each and with the bodily systems DDS (5) and DGS (3) standing out. In other communities this species is mainly reported for Undefined Afflictions and Aches, antiseptic, general pain, and tiredness. A study with this species demonstrates an anti-*Helicobacter pylori* activity that may be related to its indication for the TSG body system [88]. There are records of the* M. peruiferum* activity against* Streptococcus pyogenes*,* Shigella sonnei*, and* Staphylococcus aureus* [10, 88].

In this study, it is important to point out that although some species appear with low relative importance values, their pharmacological activities have already been confirmed in several studies, such as* Triplaris gardneriana* showing good antioxidant activity attributed to the presence of flavonoids [89]. The* Croton blanchetianus *essential oil demonstrated a nociceptive [90], anti-inflammatory, gastroprotective, and antimicrobial effect [91];* Lantana camara* demonstrated antibacterial activity [9].

### 3.4. Use and Agreement of Use of Medicinal Plants

The medicinal species of the studied area were indicated for the treatment of 49 diseases, associated with 15 body systems categories. Of these, 21% had an ICF ≥ 0.50 and 26% with ICF < 0.50, and 53% did not present consensus among the informants (Table 3). None of the categories indicated here reached the maximum Informants Consensus Factor, ICF = 1. The highest values for the Informants Consensus Factor (ICF) were for the categories related to Respiratory System Disorder (0.70), Blood and Hematopoietic Organ Diseases (0.60), and Diseases of the Endocrine Glands, Nutrition and Metabolism (0.50).

For all the analyzed areas, a total of 210 diseases were mentioned, encompassed in 17 body systems, among which the Respiratory System Disorder with an ICF = 0.91 (369 citations), the Digestive System Disorder with an ICF = 0.88 (255 citations), and Injuries, Poisonings and other Consequences of External Causes (139 citations) with an ICF = 0.86 (Table 3). As observed, there is agreement in the Respiratory System Disorder (0.70/0.91) with values close to those observed in the other studied areas. The Respiratory and Digestive Disorders are the body systems with great incidence in the population, thus being the most frequently treated by the population of the semiarid and being highlighted in several ethnobotanical surveys in the northeast [35, 58, 60, 92].

The Respiratory System Disorder category with an ICF = 0.70 showed the highest number of therapeutic use (7), as well as the largest number of species used (14), giving a total of 45 citations of uses, with* Poincianella pyramidalis* (10 citations),* Anadenanthera colubrina* (8 citations), and* Amburana cearensis* (7 citations) being the most frequently used species. This system appears in the analyzed areas with the highest ICF value (0.91), the highest citations (369), and the second largest number of species (33), losing only for DND. Among the most cited diseases for this category are influenza (105) and cough (104). In this context there is similarity in the species used, since in the survey and in the community the species* A. colubrina* is used the most by the populations. However, the survey also highlighted the* A. cearensis* species used to treat respiratory diseases. Antimicrobial and antiproliferative [93] and antinociceptive and antioxidant activities [94] have been reported for the species* A. colubrina*; however, no study was carried out to verify the activity of this species on the respiratory system was observed, whereas for the species* A. cearensis* in the study by Leal et al. (2000) the bronchodilator potential of this species was demonstrated, thus confirming its efficacy for diseases affecting the respiratory (expectorant) system [95].

Systems related to Undefined Afflictions and Aches (NDDP) (ICF = 0.37), Injuries, Poisonings and other Consequences of External Causes (IPOCEC) (ICF = 0.43), Disorder of the Digestive System (DDS) (ICF = 0.40), and Disorder of the Genitourinary System (DGS) (ICF = 0.37) presented an Informants Consensus Factor <0.50. However, for the analyzed areas these categories stand out among the highest ICF values, with ICF = 0.86, ICF = 0.86, ICF = 0.88, and ICF = 0.80, respectively, showing that they are well reported by these populations. Although there are differences between the body system indications for the community in relation to the other localities in the northeast, these may be related to the lower availability of plants as therapeutic resources.

Among the aforementioned categories, the digestive system excels, being described for 32 diseases, where the most recurrent problems are stomach disorders (32), kidney problems (30), gastritis (27), liver problems (21), diarrhea (20), and toothache (20), with the most commonly used medicinal plants to treat these problems being* Myracrodruon urundeuva*,* Poincianella pyramidalis*,* Ziziphus joazeiro*, and* Ximenia americana. *For these species, there is already evidence of their potential for stomach problems:* Myracrodruon urundeuva* shows antiulcer properties [80, 81].* Poincianella pyramidalis* has been shown to be a gastroprotector [96] whose mechanism of action involves a reduction of the endogenous hydrogen sulphate content and reduction of the inflammatory process [97]. Although there is no direct evidence for the gastroprotector activity of* Ximenia americana* and* Ziziphus joazeiro*, its use by the community for this therapeutic purpose may correlate with its anti-inflammatory, cicatrizing, and antioxidant potential [96, 98, 99]. Studies have shown that* Tocoyena formosa* has anti-inflammatory [100], antinociceptive [101], and gastroprotective activities [102].

Undefined Afflictions and Aches had an ICF = 0.85 for the areas analyzed. This category generally presents high Informants Consensus Factor for Caatinga areas, such as in the work of de Oliveira et al. (2010) [54] with an ICF = 0.85 and Santos et al. (2012) [52] with an ICF = 0.77, as well as in Cerrado areas as seen in Ribeiro et al. (2014) [6] with an ICF = 0.70 and Macêdo et al. (2015) [37] with an ICF = 0.75. In the analyzed areas, it can be verified that this system holds the largest number of species used (34) and is indicated for the treatment of 25 diseases. General inflammation obtained the highest number of citations (107), with* M. urundeuva* being the species most used to treat this disease, whose activity has already been described in the literature [81, 103–105].

Genitourinary System Disorder (TSG) and Injuries, Poisonings and Other Consequences of External Causes (LEOCCE) showed, in general, a high agreement between the communities with an ICF = 0.80 and ICF = 0.86, respectively. In the majority of ethnobotanical studies with consensus among informants, these values can vary from 0.21 to 0.86 [32, 35] for TSG and from 0.48 to 0.85 [6, 45] for LEOCCE. Of the reviewed species,* Ximenia americana* was noted for presenting the second largest number of citations for LEOCCE, with scarring (12) and wounding (9), whose potential that was demonstrated by Marinho et al. (2013) [106] stood out. The genitourinary system is notable for the indication of* M. urundeuva* for diseases such as uterine inflammation (7), gynecological problems (5), and ovarian inflammation (6), whose therapeutic potential has already been proven [81, 107, 108].

The categories of Mental and Behavioral Disorders (MBD), Infectious and Parasitic Diseases (IPD), Diseases of the Skin and Subcutaneous Cellular Tissue (DSSCT), Diseases of the Musculoskeletal System and Connective Tissue (DMSCT), Neoplasm (N), Diseases of the Circulatory System (DCS), Diseases ofthe Nervous System (DNS), and Disorders of the Visual Sensory System-Eyes (DVSS-E) obtained an ICF = 0, which means that informants do not agree with the use of the species in the treatment of diseases within these categories or that they do not share information on the use of a particular species. However, in the survey for other areas in the northeast, these body systems presented an ICF that varied between 0.5 and 0.83. The TSS (OLH), N, and DIP categories in most of the Caatinga studies appear with an ICF = 0 [7, 16, 69], not occurring in disjointed Cerrado areas where the category Neoplasia ([34] (ICF = 0.77), [36] (ICF = 0.75), [39] (ICF = 0.77)), and Infectious and Parasitic Diseases ([34, 36] (ICF = 1.0) and [39] (ICF = 0.81)) stand out with a higher concordance in use among the informants.

Other categories such as DMC, DPTCS, DSOTC, TSC, and TSN also presented an ICF = 0.0 in the studied area; however, they present, for the other areas, ICF values of 0.71, 0.83, 0.65, 0.65, and 0.58, respectively. In the semiarid DMC, TSC, and TSN area, these commonly present ICF ≥ 0.50 [6, 7, 16, 35, 37, 40, 42, 52]. Only the body system category Sensory System Disorder (ear) was not present in the studied area and also did not present a consensus among the informants in the other areas (ICF = 0.0) present in the survey. This may indicate that the population of the semiarid region is poorly affected by diseases related to this category or does not present knowledge of plants that can cure these diseases.

In this study, it was demonstrated that although there are divergences of information, the knowledge of the therapeutic potential of natural products within the Quincuncá community and the information collected demonstrates its value at the level of medical knowledge, since some of the cited categories also appear with highlights in ethnobotanical studies in semiarid areas. It was also demonstrated that part of the therapeutic potential used in the community is scientifically validated by pharmacological assays.

## 4. Conclusion

Our results showed that there are expressive numbers of medicinal plant species known in the Caatinga region of northeastern Brazil, although there are divergences in their indications. The results also reinforce the importance of ethnopharmacological studies as an important criterion for the selection of plants for more detailed studies on their pharmacological and biological activity. The results also show that medicinal species such as* Myracrodruon urundeuva*,* Amburana cearensis*,* Anadenanthera colubrina*,* Libidibia ferrea*,* Ximenia americana*, and* Ziziphus joazeiro* appear to be widely distributed since they appear in most communities. The species* Myracrodruon urundeuva*,* Mimosa tenuiflora*,* Ximenia americana*, and* Amburana cearensis* stood out as they presented a great number of therapeutic indications reaching the greatest versatilities, acting in several body systems. Although some species show few therapeutic indications and body systems, these should not be disregarded for bioprospecting, since some of these have already been pharmacologically validated. Among the divergent species* Geoffroea spinosa*,* Lantana camara*,* Senegalia tenuifolia*, and* Licania rigida* can be highlighted, presenting in the community diseases that were not verified for other areas. It was possible to show that most of the semiarid medicinal species are indicated for the respiratory system, digestive system, and undefined affections and pains, influenced by diseases such as influenza, stomach problems, and general inflammation. However, although there is a divergence between the therapeutic indications of the species for the northeastern areas, the informants' consensus stands out. In this sense, these contradictions reinforce the importance of pharmacological studies of popular indications.

## Figures and Tables

**Table 1:** List of native medicinal species indicated by the interviewees of Quincuncá district and therapeutic indications cited for different communities analyzed.

Family/scientific name/vernacular name	Therapeutic indications in the community of Quincuncá	Part used/preparatio, the community of Quincuncá	NI^1^	Therapeutic indication in other areas	Part used/preparation, in other areas	NI^2^	HN
Anacardiaceae							
*Astronium fraxinifolium* (Gonçalo Alves)	Rheumatism, wound.	Stem bark (3)/Cutting (3), cataplasm, of sauce.	2	Influenza (4), cough (3), fever (2), dysentery, stomach, kidney infection, expectorant, spine pain.	Stem inner bark (4), stem bark (2), leaf/of sauce (2), decoction, infusion, sirup.	8	8728
*Myracrodruon urundeuva* (Aroeira)	Wound (4), inflammation (3), uterine inflammation (2), influenza, menstrual bleeding, anemia, healing.	Stem bark (13), Stem inner bark (5), Leaf/Of sauce (13), decoction (6), bathing (5), cutting.	7	Inflammation (25), healing (15), gastritis (8), wound (7), uterine inflammation (7), cough (7), ovarian inflammation (6), sore throat (5), kidneys (5), gynecological problems (5), cancer (4), bronchitis (4), gynecological inflammation (4), generalized pain (4), influenza (3), itching (3), diarrhea (3), stomach (3), abdominal cramp (2), external inflammation (2), liver (2), skin problems (2), allergy (2), ulcer (2), expectorant (2), uterine problems (2), bruise (2), external ulcer (2), acne, tumors, rheumatism, cracks in the feet, arthritis, bug bite, bacteria, mycoses, bleeding, germs, fungi, pain in the digestive organs, rheumatic fever, headache, toothache, antiseptic, infection, internal inflammation, intestine, abortion, spine pain, prostate, diphtheria, antibiotic, vaginal secretion, fracture, urinary infection, anemia, ear infections, intestinal cramps, depurative, respiratory diseases, burn, inflammation of the skin, back pains, cystitis, urethritis, gonorrhea, sores in the mouth, gingivitis, tiredness, regulate menstruation, abdominal cramp, menstrual cramps.	Stem bark (26), stem inner bark (7), leaf (5), stalk/decoction (12), maceration (7), sirup (6), infusion (5), of sauce (4), tincture (2), bathing (3), cooked, ingestion, cutting.	72	8731
Arecaceae							
*Acrocomia aculeata* (Macaúba)	Inflammation in the eye, anemia, calmative.	Fruit (3)/Ingestion (2), decoction.	3	Expectorant, generalized pain, cough, bone problems, arthritis, nerves	Fruit (2), flower, root, seed/decoction (3), maceration, sirup, juice, oil extraction.	6	nc
*Syagrus oleracea *(Catolé)	Urinary infection.	Root/Of sauce.	1	Liver, inflammation, inflammation of the urethra and bladder.	Root/decoction.	4	nc
Bignoniaceae							
*Jacaranda jasminoides* (Caroba)	Itching, urinary infection.	Stem bark/ Bathing, decoction.	2	Venereal diseases, inflammation.	Stem bark, leaf/decoction.	2	nc
Bromeliaceae							
*Bromelia laciniosa* (Croatá)	Prostate.	Leaf/Decoction.	1	Source of protein (2), tonic, hepatitis, pneumonia, influenza.	Root, fruit/decoction.	5	nc
Burseraceae							
*Commiphora leptophloeos* (Imburana de cambão)	Indigestion (2), influenza, nasal congestion, wound, stroke/	Stem bark (6), Stem inner bark/Decoction (5), of sauce (2), inhalation.	5	Influenza (6), cough (5), bronchitis (4), inflammation (3), healing (2), sinusitis, belly ache, wound, kidneys, hoarseness, gastritis, ulcer, toothache, asthma, coryza, sore throat, generalized pain, tonic.	Stem bark (10), stem inner bark, leaf, fruit, latex, flower/decoction (5), sirup (3), of sauce (2), maceration (2), infusion, bathing.	18	nc
Chrysobalanaceae							
*Licania rigida* (Oiticica)	Uterine and ovarian inflammation.	Leaf, Stem inner bark/Decoction, of sauce.	2	Diabetes (6), cholesterol (2), inflammation (2), belly ache, diarrhea, dysentery, kidneys, lose weight, hypoglycemic, itching, dandruff, mycoses.	Leaf (7), stem bark (2), root (2)/decoction (5), infusion (5), maceration (2), of sauce.	12	9729
Convolvulaceae							
*Operculina macrocarpa * (Batata de purga)	Itching, cancer, worm.	Root-tuber (6)/Maceration (2), ingestion.	3	Worm (8), laxative (4), cough (4), purgative (4), rheumatism (4), inflammation (4), influenza (4), bronchitis (3), constipation (3), depurative (2), inappetence (2), asthma (2), hemorrhoid (2), blood purifying (2), child dentition, expectorant, respiratory diseases, amoeba.	Root-tuber (10), Seed, Root, stalk/infusion (6), sirup (5), decoction, juice, ingestion, maceration.	18	nc
Euphorbiaceae							
*Croton blanchetianus* (Marmeleiro)	Menstrual bleeding, wound, belly ache.	Stalk (3), Leaf/Cataplasm (3), cutting (3), of sauce (3), decoction, maceration.	3	Stomach (4), belly ache (4), cough (2), indigestion (2), diarrhea (2), dysentery (2), diabetes, depurative, influenza, bleeding, liver, malaise, intestine.	Stem bark (9), leaf (4), stem inner bark (2), stalk, root/decoction (4), sirup (3), infusion (2), cutting (2), maceration, of sauce, juice, cataplasm.	13	10.108
*Croton conduplicatus* Kunth (Quebra-faca)	Headache, fever.	Stem bark/Decoction.	2	Influenza (2), headache (2), toothache (2), indigestion, stomach, belly ache, inflammation in the nose, sinusitis, fever.	Leaf (4), stem bark (3)/decoction (2), cataplasm, of sauce, infusion.	9	nc
*Croton heliotropiifolius* Kunth (Velame)	Influenza, cough.	Root (4)/Of sauce (3), decoction.	2	Spine pain (4), inflammation (2), influenza (2), cough (2), furuncle (2), generalized pain (2), sinusitis, malaise, indigestion, depurative, back pains, worm, tumors, fever, diarrhea, wound, dermatitis, wart, infection, blood purifying, rheumatism, stomach, lesion, internal inflammation, healing.	Root (5), leaf (5), latex (2)/decoction (3), infusion (3), cataplasm, of sauce, sirup, bathing.	25	9732
Fabaceae							
*Amburana cearensis* (Umburana de cheiro/Camaru)	Influenza (5), expectorant, sinusitis, wound.	Stem bark (11), Stem inner bark/Decoction (7), of sauce (4), inhalation (3), bathing.	4	Influenza (18), cough (13), sinusitis (10), bronchitis (7), inflammation (7), generalized pain (5), headache (4), stomach (4), healing (4), expectorant (3), sore throat (3), anorexia (3), constipation (3), external ulcer (3), urinary infection (3), tonic (2), thrombosis (2), hypertension (2), snake bite (2), stroke (2), fever (3), gastritis (2), vertigo (2), regulate menstruation (2), nasal congestion, rhinitis, muscle aches, calmative, asthma, respiratory diseases, toothache, colic on baby, nausea, colic, intestine, centipede sting, dehydration, diarrhea, migraine, vomit, joint pain, depurative, diuretic, kidney infection, coryza, inflammation of the skin, gynecological inflammation, back pains, worm, poor circulation, heart, stroke, fever, measles, cold, gynecological problems.	Stem bark (24), seed (18), fruit (5), stem inner bark (4), leaf (4), stalk/decoction (12), sirup (12), infusion (9), maceration (8), of sauce (5), bathing (3), inhalation (2), cooked, cataplasm.	56	nc
*Anadenanthera colubrina* (Angico)	Cough (4), expectorant (2), influenza (2).	Stem inner bark (6) Stem bark (5), Resin/Of sauce (11), decoction, sirup.	3	Cough (14), inflammation (9), influenza (9), bronchitis (5), asthma (3), expectorant (3), sore throat (3), whooping cough (2), stomach (2), cold (2), healing (2), wound (2), anemia (2), infection (2), insomnia, depurative, anticoagulant, bleeding, nasal congestion, belly ache, cancer, lung diseases, antiseptic, antiallergic, tuberculosis, fracture, tiredness, pulmonary edema, fortifier, respiratory diseases, swelling, fever, back pains, diarrhea, venereal diseases, muscle aches.	Stem bark (17), stem inner bark (5), fruit (3), resin (3), leaf/sirup (12), decoction (6), maceration (5), infusion (4), of sauce (3), cataplasm, bathing.	36	9311
*Bauhinia cheilantha* (Pata de vaca/Mororó)	Diabetes (2).	Leaf (2)/Decoction (2).	1	Diabetes (11), influenza (4), cough (3), kidneys (3), cholesterol (3), hypertension (4), headache (2), belly ache (2), inflammation (2), Spine pain (2), tonic (2), expectorant, depurative, indigestion, asthma, cancer, tiredness, respiratory diseases, burning in the urethra, sexual impotence, nerves, urinary infection, anemia, uterus, sore throat, stomach, blood cramps, heartburn.	Stem bark (12), leaf (11), root (4), stem inner bark (2), flower (2) seed/decoction (10), infusion (8), sirup (6), of sauce (2), cutting, maceration.	28	8729
*Enterolobium contortisiliquum* (Tamboril)	Gastritis, influenza.	Stem bark (3)/Of sauce (3).	2	Inflammation (3), scabies (2), itch, asthma, ulcer, muscle aches, worm, neuritis, sciatica, stomach, vaginal inflammation, urinary inflammation, generalized pain.	Stem bark (6), seed (2), root (2), fruit, stem inner bark/decoction (4), of sauce (3), infusion.	13	9148
*Erythrina velutina *(Mulungú)	Calmative.	Leaf/Decoction.	1	Toothache (7), insomnia (4), cough (4), worm (3), nerves (3), sinusitis (2), inflammation (2), calmative, diarrhea, healing, gynecological problems, antidepressant, diseases of the nervous system, stroke, generalized pain, menopause, poor circulation.	Stem bark (11), fruit (4), seed (2), stalk, leaf, stem inner bark/decoction (7), infusion (6), of sauce (2), sirup, bathing, maceration, cutting.	17	nc
*Geoffroea spinosa *(Umarí)	Diarrhea with blood, fever, snake bite.	Leaf (7), Fruit (3)/Decoction (9), maceration.	3	Anemia.	Stem bark/maceration.	1	9313
*Hymenaea courbaril* (Jatobá)	Anemia (4), influenza (2), wound.	Stem bark (10), Stem inner bark (2)/Decoction (6), of sauce (3), cutting (2), honey (2), bathing, ingestion, cataplasm.	3	Anemia (10), influenza (12), cough (10), bronchitis (5), prostate (4), expectorant (3), inflammation (3), kidneys (3), cancer (2), healing (2), asthma (2), generalized pain (2), gastritis (2), blood purifying, stomach, cancer (leukemia), louse, lung diseases, sore throat, lip herpes, urinary problems, fortifier, nerves, inflammation of the prostate, tiredness, pulmonary edema, bone pain, sexual impotence, inflammation of the bladder, cold, stomach, respiratory diseases, tonic, ulcer, burning in the urethra, bruise, increases blood flow, fever, headache, heartburn, urinary inflammation, poor circulation, frailty, angina, wound, constipation, nasal congestion, depurative, poisoning, blood problems, urinary infection.	Stem bark (18), fruit (11), stem inner bark (6), seed, root, sap, leaf, resin/sirup (12), decoction (9), infusion (6), maceration (5), of sauce (4), juice (2), tincture, ingestion.	51	9723
*Libidibia ferrea *(Pau-ferro/Jucá)	Toothache, urinary infection, prostate.	Stem bark (5)/Decoction (3), cataplasm (2).	3	Kidneys (9), inflammation (8), influenza (7), healing (4), cough (4), diabetes (3), generalized pain (3), knock (3), anemia (2), fracture (2), leg pain (2), spine pain (3), stroke (2), bone pain (2), heartburn (2), diarrhea (3), concussion (2), lung diseases, wound, hoarseness, indigestion, gynecological inflammation, strengthens and cleanses blood, headache, toothache, tonic, depurative, urinary infection, bronchitis, gastritis, inflammation in the urethra, bruise, rheumatism, gynecological problems, liver, edemas, respiratory diseases, stomach, vesicle, internal and external inflammation, asthma, shake, bleeding, infection, labyrinthitis.	Stem bark (12), fruit (11), leaf (3), seed (3), stalk (2), stem inner bark (2)/decoction (10), maceration (6), sirup (5), of sauce (4), immersed in cachaça or wine, mixed in milk, infusion, tincture.	46	10.106
*Mimosa caesalpiniifolia *(Sabiá)	Cough (2), influenza (2).	Stem bark (6)/Of sauce (6), decoction.	2	Inflammation (2), stroke, healing, cholesterol, gastritis, cough.	Stem bark (2)/decoction, maceration.	6	12.857
*Mimosa tenuiflora *(Jurema-preta)	Toothache (3), wound (2), urinary infection, inflammation, hemorrhoid, wound disinfection, healing.	Stem bark (12), Stem inner bark (3)/Decoction (9), cataplasm (5), bathing (4), of sauce (2), cutting.	7	Wound (9), inflammation (5), toothache (4), healing (4), cough (2), bronchitis (2), inflammation in the tooth, antiseptic, belly ache, liver influenza, diabetes, cholesterol, itch, knock, psoriasis, gynecological inflammation, generalized pain, external inflammation, gastritis, uterine inflammation, eye problem, anemia, appendicitis.	Stem bark (16), stem inner bark (3), leaf (2), flower, root/decoction (8), infusion (8), maceration (4), of sauce (3), bathing (2), cataplasm, ingestion, sirup, cutting.	24	8735
*Myroxylon peruiferum *(Balso)	Liver (2), kidneys (2), influenza, intestine, prostate, hypertension, indigestion, stomach.	Stem bark (6), Stem inner bark (3), Leaf (2)/Decoction.	8	Antiseptic, cataract, eye problem, kidneys, back pains, cough, urinary incontinence, tiredness, generalized pain, indigestion.	Stem bark (4), root, resin/decoction (2), maceration, infusion, bathing, sirup.	10	nc
*Poincianella pyramidalis *(Catingueira)	Influenza (6), cough (4), bleeding, belly ache.	Flower (12), Stem bark (2), Cataplasm (2)/Decoction (7), of sauce (4), cutting, honey.	5	Cough (10), influenza (7), diarrhea (5), bronchitis (4), stomach (6), inflammation (3), expectorant (4), belly ache (3), aphrodisiac (2), dysentery (3), respiratory diseases (2), diabetes (2), healing (2), indigestion (2), gastritis (3), prostate (2), generalized pain (2), swelling of the belly, rheumatism, nerves, sexual impotence, fortifier, cold, gynecological problems, headache, toothache, intestine, anticoagulant, fever, worm, bleeding, inflammation in the body.	Stem bark (14), flower (14), leaf (2), root, fruit/decoction (9), sirup (8), infusion (6), of sauce (2), maceration (2), juice (2), stalk, cataplasm, cutting.	32	9731
*Senegalia tenuifolia *(Unha-de-gato)	Diabetes, cancer, hypertension.	Root/Decoction, of sauce.	3	Spine pain (2), arthritis, influenza, healing, rheumatism.	Stem inner bark, fruit/decoction.	5	10.109
*Senna alexandrina *(Sena)	Constipation, indigestion.	Leaf (3)/ Decoction (3).	2	Cough, stop menstruation, lose weight.	Leaf/ decoction, of sauce.	3	nc
*Stryphnodendron rotundifolium* (Barbatimão)	Wound.	Stem bark (2)/Decoction (2).	1	Uterine inflammation (4), cancer (4), healing (4), inflammation (4), wound (2), sore throat (2), gastritis (2), ulcer (2), gynecological problems, inflammation of the skin, respiratory tiredness, diabetes, venereal diseases, bleeding, antihelminthic, hypertension, anemia, liver, furuncle, urinary infection, generalized pain, tuberculosis, vaginal infection, blood infections.	Stem bark (4), stem inner bark (3)/Of sauce (3), infusion (2), decoction (2), tincture, sirup.	24	nc
Malvaceae							
*Ceiba glaziovii* (Barriguda)	Cyst (2).	Stem inner bark (5)/Of sauce (5).	2	Edema (4), rheumatism (3), spine pain (2), influenza, anemia, inflammation, diabetes, generalized pain, inflammation of the prostate.	Stem bark (5), leaf (3), stem inner bark (2)/decoction (5), of sauce (2), sirup.	9	8654
OLACACEAE							
*Ximenia americana* (Ameixa)	Wound (2), wound disinfection, gastritis, mouth ulcers, injuries in diabetics.	Stem bark (7)/Cataplasm (4), decoction (2), cutting (2), of sauce, bathing.	5	Inflammation (13), healing (12), wound (9), uterine inflammation (4), sore throat (4), kidneys (4), gastritis (4), diabetes (3), gynecological inflammation (3), spine pain (3), generalized pain (3), anemia (2), ulcer (2), burn (2), constipation (2), stomach (2), furuncle (2), ovarian inflammation (2), liver (2), infection (2), diarrhea (2), blood purifying (2), cancer (2), itching (2), shortness of breath, antiseptic, internal inflammation (2), toothache, menstrual cramp, uterine problems, abortion, urinary infection, fracture, fortifier, stomach, gynecological problems, intestine, vaginitis, tiredness, influenza, fever, headache, edema, indigestion, prostate cancer, obesity, cough, hoarseness, venereal diseases, osteoporosis, heartburn, rheumatism, bone pain.	Stem bark (20), stem inner bark (7), leaf (2), root, fruit/infusion (8), decoction (7), maceration (5), of sauce (4), cataplasm (3), sirup (3), bathing (2).	49	nc
Polygalaceae							
*Bredemeyera brevifolia* (Laça-vaqueiro)	Diarrhea in children, diarrhea, rheumatism.	Root (2), Leaf/Decoction (3).	3	Kidneys (2), influenza, rheumatism, spine pain, gastritis.	Stem bark (2), root (2), leaf/of sauce (2), decoction (2), infusion.	5	nc
Polygonaceae							
*Triplaris gardneriana* (Croaçú)	Spine pain.	Stem inner bark/Of sauce.	1	Gastritis (2), ulcer (2), inflammation of internal organs, cancer, cough, generalized pain, heartburn, influenza, rheumatism.	Stem bark (3)/sirup (2), of sauce, decoction, infusion.	9	8732
Rhamnaceae							
*Ziziphus joazeiro* (Juazeiro)	Expectorant, dandruff.	Stem bark (3)/Cutting (3).	2	Dandruff (13), influenza (9), cough (5), tooth treatment (5), inflammation (5), antiseptic bucal (5), hair tonic (3), indigestion (3), anticary (3), stomach (2), seborrhea (2), wound (2), belly ache (2), asthma (2), healing (2), scalp problems, rheumatism, fever, antiseptic, heartburn, burn, worm, toothache, cold, hemorrhoid, bronchitis, antacid, liver, itching, pulmonary infection, anemia.	Stem bark (18), leaf (11), stem inner bark (6), fruit (4), root (2), stalk/decoction (10), maceration (8), infusion (6), cutting (5), sirup (4), of sauce (4), ingestion (3), juice, bathing.	31	9727
Rubiaceae							
*Coutarea hexandra *(Quina-quina)	Fever (2), headache, sinusitis, uterine inflammation.	Stem bark (6), Stem inner bark (2), Latex/ Decoction (4), cutting (3), infusion (2), bathing.	4	Influenza (3), sinusitis (3), inflammation (2), fever (2), lung diseases, expectorant, headache, menstrual disorders, abortion, respiratory diseases, rheumatism, inflammation in the tooth, mouth inflammation, cough.	Stem bark (8), root (3), leaf (3), stem inner bark (2)/decoction (5), infusion (4), sirup, of sauce.	14	12.859
*Genipa americana* (Jenipapo)	Fracture.	Stem bark (2)/Decoction (2), cutting.	1	Anemia (3), fracture (3), bruise (2), expectorant (2), inflammation, torsion, healing, asthma, tuberculosis, cholesterol, stomach, regulate menstruation, diabetes, nerves, osteoporosis, tonic, cough, cancer.	Fruit (6), stem bark (4), stem inner bark/Ingestion (3), cataplasm (3), decoction (2), juice, cutting, infusion, sirup.	18	8727
*Guettarda angelica * (Angélica brava)	Asthma.	Root/Decoction.	1	Constipation (2), menstrual cramp (2), fever (3).	Root (3), stem bark (2), fruit/decoction (4), infusion (2), sirup.	3	nc
*Tocoyena formosa *(Jenipapinho)	Fracture, injury to the feet and hands.	Stem bark (2), Stem inner bark/Cutting (3), cataplasm (3).	2	Rheumatism (3), bruise (4), fracture (3), torsion (3), knock, swelling, healing, luxation.	Stem bark (4), leaf (4), stem inner bark (2), latex/cataplasm (3), maceration (3), infusion, of sauce, sirup.	8	9312
Solanaceae							
*Solanum paniculatum* (Jurubeba)	Liver.	Root, Fruit/Of sauce (2).	1	Liver (11), anemia (8), tuberculosis (6), diuretic (3), cough (3), influenza (3), inflammation (2), bronchitis (2), diabetes (2), tonic (2), pneumonia (2), gastritis (2), wound, inflammation of the spleen, cirrhosis, cholesterol, circulatory problems, worm, lose weight, kidneys, respiratory diseases, vesicle, expectorant, hepatitis, nasal congestion, inflammation in the tooth, hypertension	Root (10), fruit (11), leaf (5), seed, flower, stem bark/decoction (7), juice (4), infusion (4), sirup (3), maceration (2).	27	8726
Verbenaceae							
*Lantana camara *(Camará)	Hypertension.	Leaf (3)/Decoction (3).	1	Cough (4), influenza (3), expectorant (2), rheumatism (2), fever (2), increases menstrual flow, respiratory diseases, generalized pain, belly ache.	Leaf (7), flower (4), root (3), seed/decoction (6), sirup (3), infusion (2), bathing.	9	9314
*Lippia microphylla* (Alecrim-do-mato)	Influenza (2), expectorant, sore throat.	Leaf (3)/Decoction (3), inhalation.	3	Respiratory diseases (3), antiseptic, influenza, headache, sinusitis, cough, heart, hypertension, muscle aches.	Leaf (4)/decoction (3), sirup (2), infusion, whole plant.	9	9726

HN: herbarium number; NI^1^: number of indications for the community of Quincuncá; NI^2^: number of indications; NC: number of collection in process by herbarium.

**Table 2:** Common medicinal species for the community of Quincuncá and other areas analyzed, with respective values of relative importance and number of body systems.

Family and scientific name	RI^1^	RI^2^	BS^1^	BS^2^
Anacardiaceae				
*Astronium fraxinifolium *	0.65	0.43 (0.23–0.82)	DMSCT, IPOCEC	RSD (8), DDS (3), NDDP (2), DMSCT,
*Myracrodruon urundeuva *	1.87	1.35 (0.40–2.00)	IPOCEC (5), NDDP (3), DGS (3), DBHO, RSD	NDDP (37), DGS (34), DDS (30), IPOCEC (26), RSD (20), DSSCT (9), IPD (6), DMSCT (5), N (5), DCS (2), DNS, DBHO, DASS-E
Arecaceae				
*Acrocomia aculeata*	0.97	0.61 (0.34–0.90)	DVSS-E, DBHO, MBD	RSD (2), DMSCT (2), NDDP, DNS
*Syagrus oleracea*	0.32	0,52 (0.36–0.68)	DGS	DGS (2), DDS, NDDP
Bignoniaceae				
*Jacaranda jasminoides*	0.65	0.61	DSSCT, DGS	IPD, NDDP
Bromeliaceae				
*Bromelia laciniosa*	0.32	0.50 (0.23–0.65)	DGS	NDDP (2), DEGNM (2), RSD (2)
Burseraceae				
*Commiphora leptophloeos*	1.45	0.51 (0.17–1.04)	RSD (2), DDS, IPOCEC, DNS	RSD (20), NDDP (6), DDS (3), IPOCEC (3), DGS
Chrysobalanaceae				
*Licania rigida*	0.45	0.48 (0.29–0.83)	DGS (2)	DEGNM (10), DDS (3), DSSCT (3), NDDP (2), DGS
Convolvulaceae				
*Operculina macrocarpa *	0.97	1.04 (0.22–2.00)	DSSCT, N, IPD	RSD (16), DDS (9), IPD (9), NDDP (7), DMSCT (4), DGEMN (2), DCS (2), IPOCEC, DBHO
Euphorbiaceae				
*Croton blanchetianus *	0.97	0.67 (0.30–1.32)	DGS, IPOCEC, DDS	DDS (16), RSD (3), NDDP (2), DEGNM, IPOCEC
*Croton conduplicatus *	0.65	0.68 (0.61–0.73)	DNS, NDDP	DDS (5), RSD (4), DNS (2), NDDP
*Croton heliotropiifolius *	0.45	1.07 (0.17–2.00)	RSD (2)	NDDP (8), DSSCT (6), RSD (5), DDS (4), DMSCT (4), IPD, N
Fabaceae				
*Amburana cearensis *	0.90	1.33 (0.58–2.00)	RSD (7), IPOCEC	RSD (59), NDDP (15), DDS (14), DCS (9), DGS (8), DNS (8), NDDP (7), IPOCEC (6), DEGNM (3), IPD (3), DMSCT (3), DSSCT (2), MBD
*Anadenanthera colubrina *	0.57	0.92 (0.17–1.66)	RSD (8)	RSD (45), NDDP (17), IPOCEC (6), DDS (5), DBHO (3), IPD (2), DMSCT (2), MBD, N
*Bauhinia cheilantha *	0.32	1.17 (0.28–2.00)	DEGNM (2)	DGEMN (15), RSD (10), DDS (8), NDDP (7), DGS (6), DNS (3), DCS (3), DMSCT (2), IPOCEC, N, SI, DBHO
*Enterolobium contortisiliquum*	0.65	0.52 (0.24–0.77)	RSD, DDS	NDDP (5), IPD (2), DSSCT (2), RSD, DNS (2), DMSCT, DDS, DGS
*Erythrina velutina *	0.32	0.99 (0.30–1.85)	MBD	DDS (8), MBD (6), RSD (5), DNS (5), IPD (3), NDDP (3), DGS (2), IPOCEC, DCS
*Geoffroea spinosa*	0.97	0.30	DDS, NDDP, IPD	DBHO
*Hymenaea courbaril *	0.97	1.15 (0.36–2.00)	DBHO (4), RSD (2), IPOCEC,	RSD (39), DGS (12), NDDP (9), DDS (8), IPOCEC (6), DBHO (6), DBHO (5), DGEMN (4), N (3), DCS (3), DNS (2), IPD, DMSCT, SI
*Libidibia ferrea*	0.77	0.96 (0.24–2.00)	DGS (2), DDS	NDDP (16), RSD (16), IPOCEC (14), DDS (14), DGS (10), DMSCT (9), DEGNM (6), DBHO (2), DNS (2), DASS-E, MBD
*Mimosa caesalpiniifolia*	0.45	0.65 (0.17–1.62)	RSD (4)	NDDP (2), IPOCEC, DNS, DGEMN, DDS, RSD
*Mimosa tenuiflora *	1.87	0.68 (0.22–1.32)	IPOCEC (4), DDS (3), DGS, DCS, NDDP	IPOCEC (11), NDDP (11), DDS (9), RSD (5), DEGNM (3), DSSCT (2), DGS (2), DVSS-E
*Myroxylon peruiferum *	1.80	0.60 (0.17–1.12)	DDS (5), DGS (3), RSD, DCS	NDDP (3), DMSCT (2), TSS (OLH-2), DDS (2), RSD, DGS
*Poincianella pyramidalis*	1.10	1.00 (0.30–2.00)	RSD (10), DDS, NDDP	RSD (26), DDS (23), NDDP (8), SI (3), DEGNM (3), DGS (3), IPOCEC (2), DNS (2), DCS, DMSCT, DBHO, IPD
*Senegalia tenuifolia*	0.97	0.54 (0.44–0.66)	DEGNM, N, DCS	DMSCT (4), RSD, IPOCEC
*Senna alexandrina *	0.45	0.34 (0.32–0.36)	DDS (2)	RSD, DGS, DEGNM
*Stryphnodendron rotundifolium *	0.32	1.47 (0.97–2.00)	IPOCEC	DGS (7), NDDP (7), DDS (5) IPOCEC (4), N (4), IPD (3), DSSCT (2), DBHO (2), RSD, DCS, DEGNM
Malvaceae				
*Ceiba glaziovii*	0.32	0.56 (0.17–1.32)	DDS (2)	DMSCT (6), NDDP (3), IPOCEC (4), DBHO, DEGNM, N, DGS, RSD
Olacaceae				
*Ximenia americana*	1.02	1.29 (0.41–2.00)	IPOCEC (4), DDS (2)	NDDP (26), IPOCEC (24), DDS (21), DGS (17), RSD (7), DSSCT (7), DMSCT (6), DGEMN (5), DBHO (3), N (3), DNS, IPD
Polygalaceae				
*Bredemeyera brevifolia *	0.77	0.29 (0.17–0.44)	DDS (2), DMSCT	DDS (3), DMSCT (2), RSD
Polygonaceae				
*Triplaris gardneriana *	0.32	0.77 (0.17–1.55)	DMSCT	NDDP (3), DDS (3), RSD (2), N, DMSCT
Rhamnaceae				
*Ziziphus joazeiro *	0.65	1.05 (0.42–2.00)	RSD, DSSCT	DDS (22), DSSCT (21), RSD (21), NDDP (7), IPOCEC (2), DMSCT, IPD, DCS, DBHO
Rubiaceae				
*Coutarea hexandra *	1.30	0.57 (0.44–1.16)	NDDP (2), RSD, DNS, DGS	RSD (10), NDDP (3), DGS (2), DDS (2), DMSCT, DNS
*Genipa americana*	0.32	0.66 (0.25–2.00)	IPOCEC	IPOCEC (10), RSD (4), DEGNM (3), DMSCT (2), DBHO (2), IPD, DDS, DNS, DGS, NDDP (2), N
*Guettarda angelica *	0.32	0.80 (0.30–1.02)	RSD	NDDP (4), DDS (3), DGS (3)
*Tocoyena formosa*	0.65	0.34 (0.24–0.55)	IPOCEC (2)	IPOCEC (11), DMSCT (2), DSSCT
Solanaceae				
*Solanum paniculatum *	0.32	0.98 (0.30–1.75)	DDS	DDS (15), RSD (13), DBHO (7), IPD (7), NDDP (5), DGS (4), DEGNM (4), DCS (2), IPOCEC
Verbenaceae				
*Lantana camara *	0.32	0.58 (0.22–2.00)	DCS	DCS, RSD (4), DMSCT
*Lippia microphylla *	0.57	0.82 (0.32–1.66)	RSD (4)	RSD (14), NDDP (4), DCS (2), DMSCT (2), DNS, DDS

BS^2^: body systems (number of citations) from other areas; DASS-E: Disorders of the Auditory Sensory System-Ears; DBHO: Diseases of Blood and Hematopoietic Organs; DCS: Diseases of the Circulatory System; DDS: Disorder of the Digestive System; DEGNM: Disease of the Endocrine Glands, Nutrition and Metabolism; DGS: Disorder of the Genitourinary System; DMSCT: Diseases of the Musculoskeletal System and Connective Tissue; DNS: Diseases of the Nervous System; DSSCT: Diseases of the Skinand Subcutaneous Cellular Tissue; DVSS-E: Disorders of the Visual Sensory System-Eyes; IPD: Infectious and Parasitic Diseases; IPOCEC: Injuries, Poisonings and other Consequences of External Causes; MBD: Mental and Behavioral Disorders; N: neoplasms; BS^1^: body systems (number of citations) community of Quincuncá; NDDP: nondefined disorders or pain; RI^1^: values of relative importance for the community of Quincuncá; RI^2^: averages, minimum, and maximum values, range of values of relative importance for other areas; RSD: Respiratory System Disorder; SI: sexual impotence.

**Table 3:** Factor of informant consensus based on the use of medicinal species by informants from Quincuncá community and citations of uses in other areas.

Body systems/therapeutic purposes/(number of citations) community of Quincuncá	NS^1^	IFC^1^	Body systems/therapeutic purposes (number of citations)/other areas	IFC^2^	NS^2^
*NDDP*: inflammation (4), fever (4), bleeding.	(6) *Myracrodruon urundeuva*, *Mimosa tenuiflora*, *Croton conduplicatus*, *Geoffroea spinosa*, *Coutarea hexandra*, *Poincianella pyramidalis*.	0,37	*NDDP* (25–236): inflammation (107), generalized pain (30), fever (17), tonic (11), ulcer (11), depurative (10), bleeding (6), infection (6), antiseptic (6), tiredness (5), internal inflammation (5), external inflammation (4), swelling (3), malaise (2), vertigo (2), allergy (2), antiallergic, inflammation in the body, antacid, shake, frailty, colic, colic on baby, antibiotic, hepatitis	0,85	(34)* Syagrus oleracea, Acrocomia aculeata, Jacaranda jasminoides, Bromelia laciniosa, Commiphora leptophloeos, Licania rigida, Operculina macrocarpa, Croton blanchetianus, Croton conduplicatus Croton heliotropiifolius, Anadenanthera colubrina, Bauhinia cheilantha, Enterolobium contortisiliquum, Erythrina velutina, Mimosa caesalpiniifolia, Mimosa tenuiflora, Myroxylon peruiferum, Poincianella pyramidalis, Stryphnodendron rotundifolium, Ceiba glaziovii, Triplaris gardneriana, Ziziphus joazeiro, Coutarea hexandra, Genipa americana, Guettarda angelica, Tocoyena formosa, Solanum paniculatum, Lantana camara, Lippia microphylla, Ximenia americana, Libidibia ferrea, Hymenaea courbaril, Amburana cearensis, Myracrodruon urundeuva*
*DCM*: calmative (2)	(2) *Acrocomia aculeata*, *Erythrina velutina*.	0,0	*DCM* (3–8): insomnia (5), calmative (2), antidepressant	*0,71*	*(3) Anadenanthera colubrina, Erythrina velutina, Amburana cearensis*
*DEGNM*: diabetes (3)	(2) *Bauhinia cheilantha*, *Senegalia tenuifolia*.	0,50	*DEGNM* (9–55): diabetes (32), cholesterol (9), fortifier (3), anorexia (3), source of protein (2), lose weight (2), inappetence (2), hypoglycemic, obesity	0,70	(17) *Bromelia laciniosa, Poincianella pyramidalis, Stryphnodendron rotundifolium, Licania rigida, Operculina macrocarpa, Croton blanchetianus, Anadenanthera colubrina, Bauhinia cheilantha*, *Mimosa caesalpiniifolia, Mimosa tenuiflora, Senna alexandrina, Ceiba glaziovii, Genipa americana, Solanum paniculatum, Ximenia americana*, *Libidibia ferrea, Hymenaea courbaril*
*IPD*: worm	*Operculina macrocarpa*.	0,0	*IPD* (11–37): worm (16), tuberculosis (10), venereal diseases (3), antihelminthic, lip herpes, measles, bacteria, gonorrhea, germs, fungi, amoeba	*0,63*	*(14) Operculina macrocarpa, Croton heliotropiifolius, Anadenanthera colubrina, Enterolobium contortisiliquum, Erythrina velutina, Poincianella pyramidalis, Jacaranda jasminoides Stryphnodendron rotundifolium, Ziziphus joazeiro, Genipa americana, Solanum paniculatum, Hymenaea courbaril, Amburana cearensis, Myracrodruon urundeuva*
*DSSCT*: itching (2), dandruff	(3) *Jacaranda jasminoides*, *Operculina macrocarpa*, *Ziziphus joazeiro*.	0,0	*DSSCT* (18–55): dandruff (14), itching (7), furuncle (5), inflammation of the skin (4), burn (4), hair tonic (3), mycoses (2), skin problems (2), dermatitis (2), scabies (2), itch (2), seborrhea (2), louse, scalp problems, dehydration, acne, psoriasis, wart	0,83	(10) *Licania rígida, Croton heliotropiifolius, Enterolobium contortisiliquum, Mimosa tenuiflora, Stryphnodendronrotundifolium, Ziziphus joazeiro, Ximenia americana*, *Hymenaea courbaril*, *Amburana cearensis, Myracrodruon urundeuva*
*DBHO*: anemia (6)	(3) *Myracrodruon urundeuva*, *Acrocomia aculeata*, *Hymenaea courbaril*.	0,60	*DBHO* (8–45): anemia (33), blood purifying (6), anticoagulant (2), blood infections, strengthens and cleanses blood, blood problems	*0,68*	*(15) Operculina macrocarpa, Ceiba glaziovii, Myracrodruon urundeuva*, *Croton heliotropiifolius, Anadenanthera colubrina, Bauhinia cheilantha, Geoffroea spinosa, Mimosa tenuiflora, Poincianella pyramidalis, Ximenia americana, Libidibia ferrea, Stryphnodendron rotundifolium, Genipa americana, Solanum paniculatum, Hymenaea courbaril*
*DMSCT*: rheumatism (2), spine pain	(3) *Astronium fraxinifolium*, *Bredemeyera brevifolia*, *Triplaris gardneriana*.	0,0	*DMSCT* (12–67): rheumatism (22), spine pain (19), bone problems (5), back pains (5), muscle aches (4), arthritis (3), osteoporosis (2), concussion (2), leg pain (2), joint pain, rheumatic fever	*0,65*	*(24) Astronium fraxinifolium, Acrocomia aculeata, Operculina macrocarpa, Croton heliotropiifolius, Anadenanthera colubrina, Bauhinia cheilantha, Enterolobium contortisiliquum*, *Myroxylon peruiferum, Poincianella pyramidalis, Senegalia tenuifolia, Ceiba glaziovii, Bredemeyera brevifolia, Triplaris gardneriana, Ziziphus joazeiro, Coutarea hexandra, Genipa americana, Tocoyena formosa, Lantana camara, Lippia microphylla, Ximenia americana, Libidibia ferrea, Hymenaea courbaril*, *Amburana cearensis, Myracrodruon urundeuva*
*IPOCEC*: wound (14), wound disinfection (2), healing (2), fracture (2), injury to the feet and hands, snake bite, injuries in diabetics.	(13) *Astronium fraxinifolium*, *Myracrodruon urundeuva*, *Commiphora leptophloeos*, *Croton blanchetianus*, *Amburana cearensis*, *Hymenaea courbaril*, *Mimosa tenuiflora*, *Genipa americana Stryphnodendron adstringens*, *Ximenia americana*, *Tocoyena formosa*, *Geoffroea spinosa*.	0,43	*IPOCEC* (14–139): healing (59), wound (35), bruise (11), fracture (11), knock (6), edemas (6), torsion (4), luxation, lesion, poisoning, snake bite, centipede sting, bug bites, cracks in the feet	0,86	(19) *Commiphora leptophloeos, Croton heliotropiifolius, Anadenanthera colubrina, Erythrina velutina, Mimosa caesalpiniifolia, Mimosa tenuiflora, Poincianella pyramidalis, Senegalia tenuifolia Ceiba glaziovii, Stryphnodendron rotundifolium, Ziziphus joazeiro, Genipa americana*, *Tocoyena formosa, Solanum paniculatum*, *Ximenia americana*, *Libidibia ferrea, Hymenaea courbaril*, *Amburana cearensis, Myracrodruon urundeuva*
*N*: cancer (2)	(2) *Operculina macrocarpa*, *Senegalia tenuifolia*.	0,0	*N* (4–20): cancer (16), tumors (2), prostate cancer, leukemia	0,57	(9) *Croton heliotropiifolius, Bauhinia cheilantha, Anadenanthera colubrina, Genipa americana,Stryphnodendron rotundifolium, Triplaris gardneriana, Ximenia americana, Hymenaea courbaril, Myracrodruon urundeuva*
*DCS*: hypertension (3), hemorrhoid.	(4) *Mimosa tenuiflora*, *Myroxylon peruiferum*, *Senegalia tenuifolia*, *Lantana camara.*	0,0	*DCS* (9–28): hypertension (9), external ulcer (5), hemorrhoid (3), thrombosis (2), poor circulation (4), heart (2), angina, increases blood flow	0,65	(10) *Bauhinia cheilantha, Erythrina velutina, Stryphnodendron rotundifolium, Ziziphus joazeiro, Operculina macrocarpa, Solanum paniculatum, Lippia microphylla, Hymenaea courbaril, Amburana cearensis, Myracrodruon urundeuva*
*DDS*: toothache (4), liver (3), indigestion (2), belly ache (2), gastritis (2), cyst (2), diarrhea with blood, diarrhea in children, intestine, stomach, constipation, mouth infection.	(13) *Commiphora leptophloeos*, *Croton blanchetianus*, *Poincianella pyramidalis*, *Enterolobium contortisiliquum*, *Ximenia americana*, *Geoffroea spinosa*, *Bredemeyera brevifolia*, *Libidibia ferrea*, *Mimosa tenuiflora*, *Myroxylon peruiferum*, *Solanum paniculatum*, *Senna alexandrina*, *Ceiba glaziovii*.	0,40	*DDS* (32–255): stomach (32), kidneys (30), gastritis (27), liver (21), diarrhea (20), toothache (20), belly ache (17), indigestion (13), constipation (11), dysentery (7), heartburn (7), intestine (6), tooth treatment (5), antiseptic bucal (5), laxative (4), purgative (4), inflammation in the tooth (3), anticary (3), vesicle (3), abdominal cramp (3), kidney infection (2), child dentition, blood cramps, appendicitis, swelling of the belly, mouth inflammation, inflammation of the spleen, cirrhosis, vomit, sores in the mouth, gingivitis, intestinal cramps	*0,88*	*(30) Astronium fraxinifolium, Syagrus oleracea, Commiphora leptophloeos, Licania rigida, Operculina macrocarpa, Croton blanchetianus, Croton conduplicatus, Croton heliotropiifolius, Anadenanthera colubrina, Bauhinia cheilantha, Enterolobium contortisiliquum, Erythrina velutina, Mimosa caesalpiniifolia, Mimosa tenuiflora, Myroxylon peruiferum, Poincianella pyramidalis, Stryphnodendron rotundifolium, Ziziphus joazeiro, Bredemeyera brevifolia, Triplaris gardneriana, Coutarea hexandra, Genipa americana, Guettarda angelica, Solanum paniculatum, Lantana camara, Ximenia americana, Libidibia ferrea, Hymenaea courbaril, Amburana cearensis, Myracrodruon urundeuva*
*DGS*: uterine inflammation (4), prostate (4), urinary infection (4), menstrual bleeding (2), ovarian inflammation, kidneys (2).	(11) *Myracrodruon urundeuva*, *Croton blanchetianus*, *Licania rigida*, *Myroxylon peruiferum*, *Coutarea hexandra*, *Syagrus oleracea*, *Jacaranda jasminoides*, *Libidibia ferrea*, *Mimosa tenuiflora*, *Bromelia laciniosa*, *Myroxylon peruiferum.*	0,37	*DGS* (29–102): uterine inflammation (17), gynecological problems (10), urinary infection (9), gynecological inflammation (9), prostate (8), ovarian inflammation (7), regulate menstruation (4), menstrual cramp (4), diuretic (4), uterine problems (4), abortion (3), inflammation of the urethra (2), inflammation of the bladder (2), venereal diseases (2), burning in the urethra (2), inflammation of the prostate (2), vaginitis, stop menstruation, menopause, urinary incontinence, vaginal infection, menstrual disorders, increases menstrual flow, urinary problems, urinary inflammation, cystitis, urethritis, vaginal secretion	*0,80*	*(21) Syagrus oleracea, Jacaranda jasminoides, Bauhinia cheilantha, Enterolobium contortisiliquum, Erythrina velutina, Mimosa tenuiflora, Myroxylon peruiferum, Poincianella pyramidalis, Senna alexandrina, Stryphnodendronrotundifolium*, *Ceiba glaziovii, Coutarea hexandra, Genipa americana, Guettarda angelica, Solanum paniculatum, Lantana camara, Ximenia americana, Libidibia ferrea, Hymenaea courbaril, Amburana cearensis, Myracrodruon urundeuva*
*DNS*: stroke, headache (2).	(3) *Commiphora leptophloeos*, *Croton conduplicatus*, *Coutarea hexandra*.	0,0	*DNS* (7–32): Headache (13), nerves (8), stroke (7), neuritis, sciatica, diseases of the nervous system, migraine	*0,58*	*(14) Acrocomia aculeata, Bauhinia cheilantha, Enterolobium contortisiliquum, Erythrina velutina, Mimosa caesalpiniifolia, Poincianella pyramidalis, Coutarea hexandra, Genipa americana, Lippia microphylla, Ximenia americana*, *Libidibia ferrea, Hymenaea courbaril*, *Amburana cearensis, Myracrodruon urundeuva*
*RSD*: influenza (24), cough (11), expectorant (5), sinusitis (2), asthma, sore throat, nasal congestion	(14) *Myracrodruon urundeuva*, *Commiphora leptophloeos*, *Croton heliotropiifolius*, *Amburana cearensis*, *Anadenanthera colubrina*, *Enterolobium contortisiliquum*, *Hymenaea courbaril*, *Mimosa caesalpiniifolia*, *Myroxylon peruiferum*, *Poincianella pyramidalis*, *Lippia microphylla*, *Ziziphus joazeiro*, *Coutarea hexandra*, *Guettarda angelica.*	0,70	*RSD* (22–369): influenza (105), cough (104), expectorant (25), pneumonia (3), bronchitis (38), asthma (15), coryza (2), sinusitis (19), hoarseness (3), respiratory diseases (12), inflammation in the nose, sore throat (20), whooping cough (2), cold (6), nasal congestion (4), lung diseases (4), pulmonary edema (2), respiratory tiredness, pulmonary infection, shortness of breath, rhinitis, diphtheria	*0,91*	*(33) Astronium fraxinifolium, Acrocomia aculeata, Bromelia laciniosa, Commiphora leptophloeos, Operculina macrocarpa, Croton blanchetianus, Croton conduplicatus, Croton heliotropiifolius, Anadenanthera colubrina, Bauhinia cheilantha*,* Enterolobium contortisiliquum, Erythrina velutina, Mimosa caesalpiniifolia, Mimosa tenuiflora, Myroxylon peruiferum, Poincianella pyramidalis, Senegalia tenuifolia, Senna alexandrina, Stryphnodendronrotundifolium, Ceiba glaziovii, Bredemeyera brevifolia, Triplaris gardneriana, Ziziphus joazeiro, Coutarea hexandra, Genipa americana, Solanum paniculatum*, *Lantana camara, Lippia microphylla, Ximenia americana, Libidibia ferrea, Hymenaea courbaril*, *Amburana cearensis, Myracrodruon urundeuva*
*DVSS-E*: inflammation in the eye	(1) *Acrocomia aculeata*.	0,0	*DVSS-E* (2-3): eye problem (2), cataract	0,50	(2) *Mimosa tenuiflora, Myroxylon peruiferum*
-	-	-	*DASS-E* (2-2): labyrinthitis, ear infections	0,0	(2) *Libidibia ferrea, Myracrodruon urundeuva*
-	-	-	*SI* (2–5): sexual impotence (3), aphrodisiac (2)	0,50	(3) *Bauhinia cheilantha, Poincianella pyramidalis, Hymenaea courbaril*

DASS-E: Disorders of the Auditory Sensory System-Ears; DBHO: Diseases of Blood and Hematopoietic Organs; DCS: Diseases of the Circulatory System; DDS: Disorder of the Digestive System; DEGNM: Disease of the Endocrine Glands, Nutrition and Metabolism; DGS: Disorder of the Genitourinary System; DMSCT: Diseases of the Musculoskeletal System and Connective Tissue; DNS: Diseases of the Nervous System; DSSCT: Diseases of the Skin and Subcutaneous Cellular Tissue; DVSS-E: Disorders of the Visual Sensory System-Eyes; ICF^1^: Informants Consensus Factor for the community of Quincuncá; ICF^2^: Informants Consensus Factor for other areas; IPD: Infectious and Parasitic Diseases; IPOCEC: Injuries, Poisonings and other Consequences of External Causes; MBD: Mental and Behavioral Disorders; N: Neoplasms; NDDP: Nondefined Disorders or Pain; NS^1^: number of species for the community of Quincuncá; NS^2^: number of species for other areas; RSD: Respiratory System Disorder; SI: Sexual impotence.
